# Adjuvant vitamin C for sepsis: mono or triple?

**DOI:** 10.1186/s13054-019-2717-x

**Published:** 2019-12-27

**Authors:** Angelique M. E. Spoelstra–de Man, Heleen M. Oudemans–van Straaten, Mette M. Berger

**Affiliations:** 1 Department of Intensive Care, Amsterdam UMC, location VUmc, De Boelelaan 1117, 1081 HV Amsterdam, the Netherlands; 2 Service of Intensive Care Medicine and Burns, Lausanne University Hospital (CHUV), Rue du Bugnon 46, CH-1011 Lausanne, Switzerland

**Keywords:** Vitamin C, Hydrocortisone, Thiamine, Sepsis

Recently, the largest randomized clinical trial (RCT) of intravenous vitamin C in septic patients until now, the CITRIS-ALI trial, showed a 16.5% lower mortality in the vitamin C group compared to placebo, while the primary endpoint deltaSOFA-score after 96 h was negative [1]. Mortality curves parted from day 2, which may be due to early circulatory stabilization by vitamin C [2]. Many comments on this trial addressed the methodological problem due to the exclusion of deceased and discharged patients from the deltaSOFA-score. Given the early mortality reduction in favor of vitamin C, recalculation with assignment of the worst SOFA score to deceased patients and the best score to discharged patients will undoubtedly show less organ dysfunction. So, the results of the CITRIS-ALI study support the promising role of adjuvant high-dose intravenous vitamin C for septic patients.

The CITRIS-ALI trial differed in two important aspects from the groundbreaking before-after study of Marik et al. which showed a 30% absolute mortality reduction, but had the limitation of not being randomized [2]. First, the i.v. dose of 200 mg/kg/day vitamin C (i.e., 16 g/day for 80 kg BW) was higher than the 6 g/day of the HAT-therapy. Second, vitamin C was administered as monotherapy and not in combination with hydrocortisone and thiamine (HAT-therapy, based on the hypothesis that hydrocortisone and thiamine have potentially synergistic effects with vitamin C). Although the positive results of the CITRIS-ALI study on mortality question the necessity of co-administration of hydrocortisone and thiamine when vitamin C is dosed sufficiently high, notably 66% of patients in CITRIS-ALI study received corticosteroids.

The rationale for vitamin C in sepsis is strong. Humans are unable to synthesize vitamin C, while animals acutely increase its production during stress to protect cells against the overwhelming production of reactive oxygen species (ROS) causing endothelial and mitochondrial injury with subsequent organ failure [3]. In animals, the endogenous vitamin C production is inversely correlated to the cortisol response. Higher cortisol responses are associated with poorer outcomes, which may be attributed to higher severity of illness, but also to vitamin C deficiency [4]. Vitamin C is our central antioxidant, which directly scavenges free radicals, prevents production of ROS, and restores other antioxidants, reducing endothelial permeability and cellular apoptosis. Furthermore, vitamin C is a neuroprotector, immunomodulator, and cofactor for synthesis of vasopressors. Increased vitamin C consumption and reduced recycling lead to abrupt hypovitaminosis C (≤ 23 μmol/l) and deficiency (≤ 11 μmol/l) as measured in 88% and 38% of the patients with septic shock [5].

The optimal dose of vitamin C is unknown, and systematic dose-finding during critical illness has not been performed. However, several facts are known. First, the antioxidant capacity of vitamin C is dose-dependent and direct radical scavenging capacity is maximal at a plasma vitamin C level > 175 mg/l (1000 μmol/l), more than ten times normal [6]. In addition, other functions like immune modulation, inhibition of bacterial replication, and neuroprotection are dose-dependent as well. Second, intravenous dosing is necessary, because enteral uptake is limited [3]. Third, intravenous vitamin C doses of 2–3 g/day are needed to normalize plasma concentrations and sustained therapy is needed to prevent reoccurrence of hypovitaminosis [7]. The dose-concentration relationship is linear, so higher doses yield proportionally higher plasma concentrations [7]. Plasma concentrations > 1000 μmol/l can be obtained with 10 g i.v. vitamin C/day [7]. In the only dose-effect study in septic patients, the higher dose of i.v. vitamin C (200 mg/kg/day) improved organ dysfunction more than the lower dose of 50 mg/kg/day. A recent meta-analysis found a reduced mortality with medium-dose vitamin C (3–10 g/day) in critically ill patients, in contrast to low (< 3 g/day) or high doses (≥ 10 g/day) [8]. However, this meta-analysis included small studies, one third of these were retrospective, duration of treatment varied, and the CITRIS-ALI was not included. So more and larger studies comparing different doses are required before recommendations can be made about the optimal dose.

Thiamine levels are reduced in 20–70% of the septic patients due to increased metabolic consumption, decreased intake, and increased loss. Low thiamine status in critically ill patients is associated with worse outcome [9]. Deficiency reduces the activity of thiamine-dependent enzymes, linked to (mitochondrial) energy production (aerobic glycolysis, Krebs cycle) and maintenance of NADPH (pentose phosphate pathway). Deficiency promotes the production of lactate and hampers the production of ATP. NADPH is essential for the maintenance of cellular pH. Thiamine is also cofactor in the production of glucose-derived neurotransmitters and of acetylcholine and myelin, thereby maintaining neuronal activity. The main role of non-phosphorylated thiamine is being an antioxidant. Furthermore, adequate thiamine status stimulates the conversion of glyoxylate into glycine instead of oxalate. This reduces the risk of oxalate nephropathy due to high-dose vitamin C [9]. The only human RCT with thiamine (200 mg/12 h) in septic shock had no impact on mortality, but post hoc analysis showed improvement of renal function with thiamine [10].

Hydrocortisone and vitamin C have several functions in common, including attenuation of nuclear factor-κB activation and subsequent generation of pro-inflammatory mediators with improvement of the endothelial barrier and microcirculatory patency. Both are necessary for the synthesis of catecholamines and improve vasopressor sensitivity. During sepsis, high amounts of ROS hamper glucocorticoid binding to its receptor and the cellular uptake of vitamin C by the sodium-vitamin C transporter2 (SVCT2). Synergy is therefore possible: vitamin C can restore glucocorticoid receptor function, and glucocorticoids can increase expression of the vitamin SVCT2 transporter [11]. In vitro, the combination showed a superior barrier-protective effect after lipopolysaccharide exposure compared to either agent alone [12].

In line with the study of Marik et al. [2], two other small retrospective studies in patients with septic shock [13] and severe pneumonia [14] showed reduced mortality with HAT-therapy, whereas another retrospective study in septic shock did not [15]. Large RCTs are currently being performed.

So, the CITRIS-ALI results support a potential role for adjuvant high-dose i.v. vitamin C for sepsis. Large RCTs are under way to confirm these results. Whether HAT-therapy induces additional beneficial effect when vitamin C is dosed sufficiently high remains to be demonstrated, although hydrocortisone and thiamine are certainly indicated in specific patient groups.

## Data Availability

Not applicable.

## Abbreviations

RCT: Randomized clinical trial
SOFA score: Sequential Organ Failure Assessment score
i.v.: Intravenous
BW: Bodyweight
HAT-therapy: Hydrocortisone-ascorbic acid-thiamine therapy
ROS: Reactive oxygen species
NADPH: Nicotinamide adenine dinucleotide phosphate
SVCT2: Sodium-vitamin C transporter 2
